# Atomically precise cluster catalysis towards quantum controlled catalysts

**DOI:** 10.1088/1468-6996/15/6/063501

**Published:** 2014-12-29

**Authors:** Yoshihide Watanabe

**Affiliations:** Quantum Controlled Catalysis Program, Frontier Research Center, Toyota Central R&D Labs. Inc., 41-1 Nagakute, Aichi, Japan

**Keywords:** cluster, size-selected, atomically precise, deposited, catalysis, surface, supported

## Abstract

Catalysis of atomically precise clusters supported on a substrate is reviewed in relation to the type of reactions. The catalytic activity of supported clusters has generally been discussed in terms of electronic structure. Several lines of evidence have indicated that the electronic structure of clusters and the geometry of clusters on a support, including the accompanying cluster-support interaction, are strongly correlated with catalytic activity. The electronic states of small clusters would be easily affected by cluster–support interactions. Several studies have suggested that it is possible to tune the electronic structure through atomic control of the cluster size. It is promising to tune not only the number of cluster atoms, but also the hybridization between the electronic states of the adsorbed reactant molecules and clusters in order to realize a quantum-controlled catalyst.

## 1. Introduction

In this article, we review the different types of reactions involving metal clusters deposited on a support material in light of the relationship between atomically controlled cluster sizes and catalytic activity. We first briefly describe size-dependent catalytic activity of free clusters for typical reactions based on the reviewed articles.

Böhme and Schwarz mentioned,

Gas-phase studies on ‘isolated’ reactants provide an ideal arena for detailed experiments of the energetics and kinetics of any bond-making and bond-breaking process at a strictly molecular level. In the last decade mass-spectrometric experiments with advanced techniques have been exploited to provide useful insight into the elementary steps of various catalytic reactions and to characterize reactive intermediates that have previously not been within reach of condensed phase techniques. Gas-phase studies will, in principle, never account for the precise mechanisms, energetics, and kinetics operating in applied catalysis. However, such experimental studies, complemented by computational investigations, are not at all without meaning, for they provide a conceptual framework and an efficient means to obtain direct insight into reactivity patterns, the role of differential ligation, the importance of aspects of electronic structure, and the nature of crucial intermediates. Furthermore, as these gas-phase studies can be performed under well-defined conditions, they play a key role in the evolution of approaches aimed at a more comprehensive understanding of elementary steps, knowledge of which is mandatory for the design of tailor-made catalysts [1].

**Figure 1.**
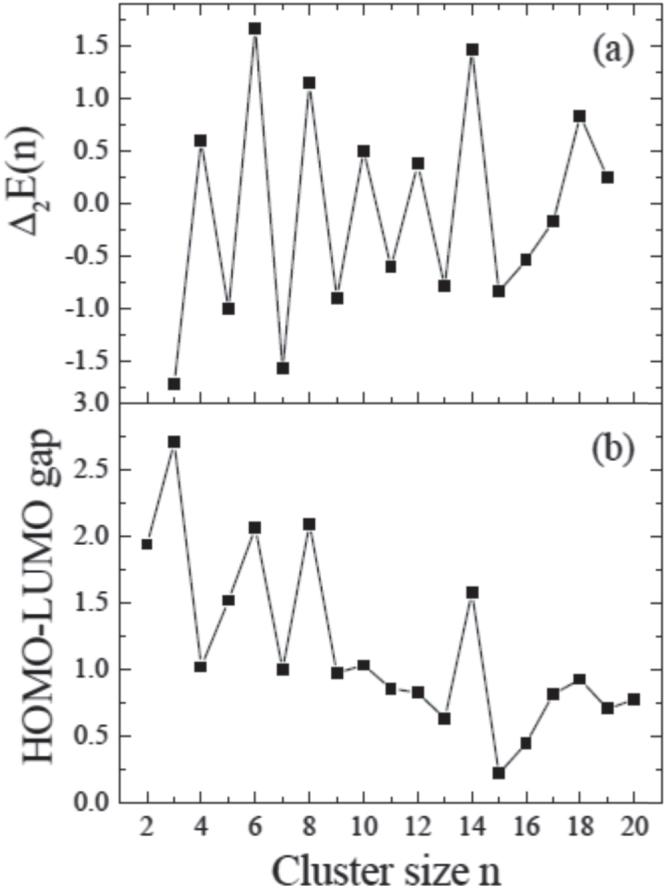
Electronic properties of gas-phase Au_*n*_ cluster properties with cluster size: (a) Second differences of cluster energies *Δ*_2_E(*n*) = E(*n* − 1) + E(*n* + 1)—2E(*n*) (eV); (b) HOMO-LUMO gaps (eV). (Reprinted with permission from [7], © 2001 American Physical Society.)

There exist ample studies reporting size-dependent reactions on gas-phase clusters. We have valuable assets on gas-phase cluster catalysis, which we should utilize. Computational studies of gas-phase clusters, including their geometry and electronic structure, have been reported [2–7]. There still exists a complex issue that is difficult to briefly review in this paper. The studies of the size-dependent electronic structures and stabilities of gas-phase clusters considering their geometries are reviewed briefly. The size-dependent electronic structures of gas-phase Au_*n*_ clusters in relationship to a highest-occupied molecular orbital–lowest unoccupied molecular orbital (HOMO–LUMO) gap are shown in figure 1 [7].

Figure 1 shows the electronic properties of the Au_*n*_ (*n* = 2−20) clusters based on density functional theory (DFT) with local density approximation [7]. Both the *Δ*_2_E(*n*) and HOMO–LUMO gap exhibit odd–even oscillations. The even-numbered Au_*n*_ clusters were relatively more stable than the neighboring odd-sized ones and have larger HOMO–LUMO gaps. This was explained as follows. The structures of the smallest gold clusters were planar and dominated by s electrons. The contributions of d electrons become more important and the structural transition from two-dimensional (2D) to three-dimensional (3D) took place at the size of seven atoms. The high stability of Au_8_ and Au_18_ can be understood by the effect of the s-electron shell. Tabular cage-like structures are preferred in the range of *n* = 10−14, and a structural transition from tabular cage-like structure to compact near-spherical structure is found around *n* = 15. The tabular configurations for the Au_*n*_ clusters with *n* = 10–14 were suggested to depend on the interplay between electronic and geometric effects [7].

A number of excellent studies on gas-phase cluster catalysis is still in progress [8–10]. However, it is beyond this paper’s scope to discuss about gas-phase isolated cluster in more detail. For more information on the gas-phase reactions of clusters, please read references [1, 11–17]. We limit the discussion of deposited (supported) atomically precise clusters that have an interaction with surface (support). We do not deal here with non-atomically precise and diameter-controlled or mono-dispersed clusters with some ligands that are typically produced by chemical synthesis, essentially. There are also numerous high-quality research projects on deposited (supported) atomically precise clusters outside the field of catalytic chemistry [18–22].

Several excellent research groups have made great effort to demonstrate specific catalytic reactions of a few clusters deposited onto a substrate [23–30]. In particular, several studies have reported size-specified catalytic activity with evidence indicating that there exists a correlation with the electronic state [31–33]. Although mechanisms for these reactions have been suggested, general guidelines for catalyst design are poorly understood.

A criticism against atomically precise clusters is that they are ‘useless in terms of practical usage’. It is impossible to maintain the cluster size because it is too small to avoid thermal aggregation at the high temperatures that are of practical interest. Such notions are deemed pessimistic to discount the capability of clusters to exhibit strong cluster–support interaction and form a surface compound.

Some researchers are targeting atomically precise large clusters. Several studies by the research group of Palmer have reported that the cluster-size-dependent (*n* = 55–400) catalytic activity/selectivity of gas-phase hydrogenation of 1-pentyne over size-selected Pd clusters supported on graphite powder [34, 35]. The challenge would be to demonstrate the thermal stability of cluster catalysts under realistic reaction conditions, which is also beyond this paper’s scope.

Here, we overview catalytic activity of metal clusters deposited on a support in light of atomically controlled sizes in relation to the type of reactions. The reactions reviewed in this paper include CO oxidation, NO–CO reactions, acetylene cyclotrimerization, hydrazine decomposition, cyclohexene aerobic oxidation, photocatalytic reactions, and electrochemical reactions (oxygen reduction reactions and oxygen evolution reactions). On a final note, the results of numerical approaches are mentioned in addition to the experimental results.

## 2. Heterogeneous reactions on atomically precise clusters supported on a substrate

The first catalytic experiment on size-selected clusters supported on a substrate was carried out by Heiz *et al* [36]. They studied the size-dependence of the reactivity of CO for oxidation by mass-selected Ni clusters deposited on a MgO(100) substrate. We will discuss the size dependence later. Note that we should distinguish the reaction rate between per atom and per cluster.

### 2.1. CO oxidation reaction

**Figure 2.**
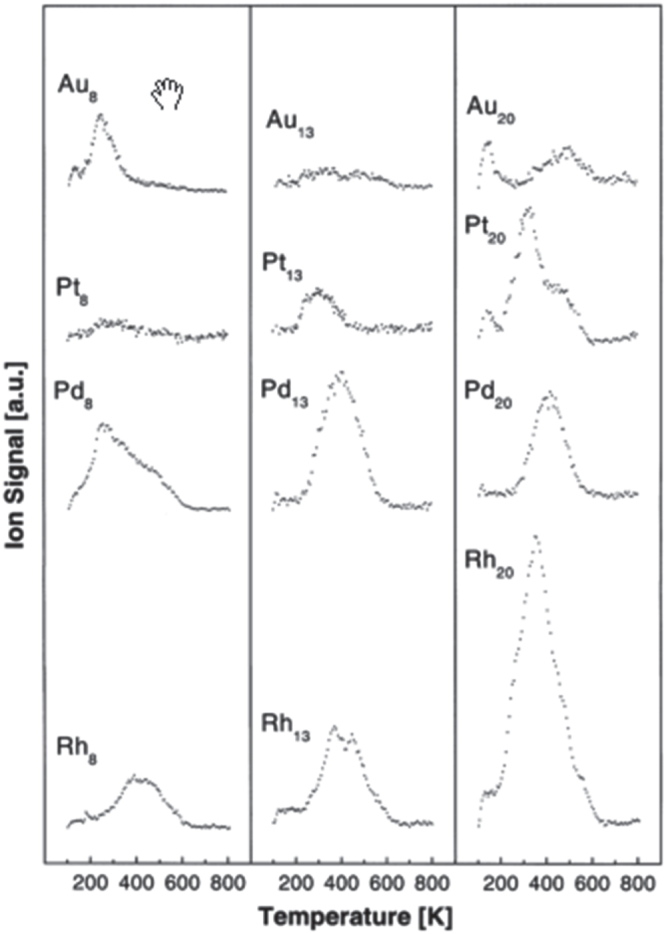
CO combustion at 350 K on Au_*n*_, Pt_*n*_, Pd_*n*_, and Rh_*n*_ (*n* = 8, 13, 20) obtained by a one-heating-cycle experiment. The model catalysts were first exposed to an average of 20 molecules of ^18^O_2_ per deposited metal atom and subsequently by the same number of carbon monoxide atoms. The CO_2_ signal was scaled to the coverage of the deposited metal clusters. (Reprinted with permission from [37], © 2000 Elsevier Science B.V.)

Heiz *et al* [37] studied the CO oxidation at 350 K by size-selected clusters of Au_*n*_, Pt_*n*_, Pd_*n*_, and Rh_*n*_ (*n* = 8, 13, 20) supported on MgO(100) as shown in figure 2. The strong size-dependence of the reaction was noted. The size dependence of produced CO_2_ intensity/atom for each element is shown in figure 2, which was derived from figure 1 for this paper. Although CO oxidation has long been considered to be structure-insensitive, the results indicated distinct size effects. For Pt_13_, Pd_13_, and Rh_13_ as well as Au_13_, exhibited different reactivities. This suggests that their electronic structure mainly governs their reactivity. Au_13_ had a low-density of states (DOS) around the Fermi level (*E*_F_), preventing coupling with the antibonding state of oxygen. On the other hand, the Pt_13_ and Pd_13_ atomic d-states overlapped *E*_F_, resulting in a high DOS and greater reactivity. For Pd_13_, an additional unoccupied shell-2p∗ state was situated at *E*_F_. Hybridization between the antibonding *π*∗_g_ state of oxygen and the cluster’s electronic states at *E*_F_ would activate adsorbed oxygen molecules. The electronic states of oxygen hybridized more efficiently with the high density of d-states of Pd and Pt clusters.

They also investigated the influence of the defect density on MgO(100) films. It was found that CO oxidation on Au_8_ at a low temperature (∼240 K) was activated after deposition on defect sites of MgO substrate. On the other hand, CO oxidation on Pd_8_ was not suppressed when adsorbed on defect-poor films. Thus, the role of these defects was to anchor clusters to the MgO(F-centres) surface to reduce agglomeration and sintering, charge transfer to the gold cluster to enhance O_2_ adsorption and to reduce the activation energy.

Charge transfer to the Pd_8_ may have less effect because the DOS around *E*_F_ is characterized by the superposition of atomic d-states; thus, subtle changes in the Pd_8_ electronic structure were less important than they were for Au_8_. It was also mentioned that the chemical properties of small metal clusters can be tuned by the size and distinct cluster–support interaction. The addition of a single Pt atom to Pt_14_ increased the reactivity of platinum clusters by a factor of three. Rh_20_ had the highest reactivity of all the investigated clusters.

Anderson *et al* studied the temperature-programmed reaction (TPR) of CO with O_2_ catalysed by size-selected Pd clusters (Pd_*n*_, for *n* = 1, 2, 4, 7, 10, 16, 20, 25) deposited on rutile TiO_2_(110) as shown in figure 4 [31]. X-ray photoemission spectroscopy (XPS) revealed that the Pd 3d binding energy (BE) varied non-monotonically with cluster size and that these changes correlated with strong size variations in CO oxidation activity. Taking final-state effects into account, the low activity was correlated with the higher-than-expected Pd 3d binding energy, which was attributed to a particularly stable valence electronic structure; electron transfer from the TiO_2_ support to the Pd clusters also occurred. Ion scattering spectroscopy (ISS) showed that small clusters formed single-layer islands on the surface, and the formation of a second layer occurred for clusters larger than Pd_10_ [31].

**Figure 3.**
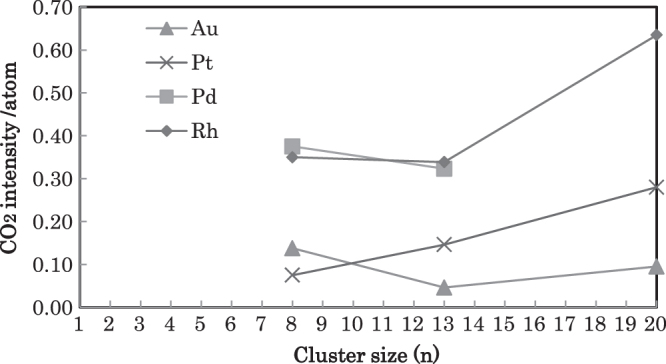
Size dependence of CO oxidation at 350 K on Au_*n*_, Pt_*n*_, Pd_*n*_, and Rh_*n*_ (*n* = 8, 13, 20)/MgO(100) reproduced from figure 2 by the author.

**Figure 4.**
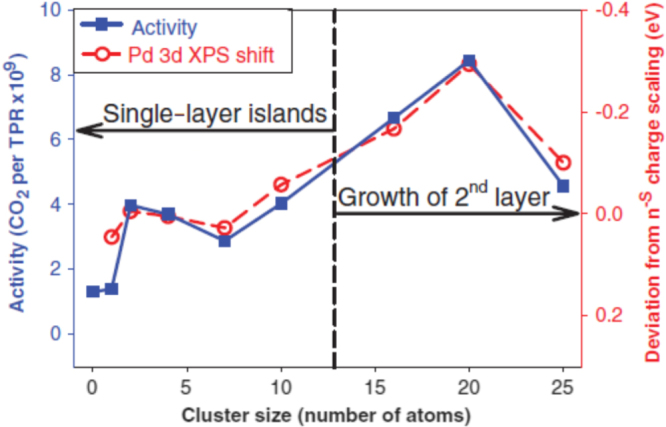
CO oxidation activity observed during TPR (left axis, solid squares) compared with shifts in the Pd 3d binding energy, relative to expectations from smooth bulk scaling (right axis, open circles), as a function of cluster size. (Reprinted with permission from [31], © 2009 by the American Association for the Advancement of Science.)

CO oxidation activity observed during TPR (left axis, solid squares) compared with shifts in the Pd 3d BE, relative to expectations from smooth bulk scaling (right axis, open circles), as a function of cluster size. The right hand axis (inverted scale) shows the deviation of the experimental Pd 3d BEs from *n*^−0.2^ scaling, while the left-hand one corresponds to the CO oxidation activity given as the number of CO_2_ product molecules desorbed from each sample during TPR. The Pd electronic structure, as manifested in core-level binding-energy shifts, was strongly correlated with CO oxidation activity. Pd_2_ to Pd_10_ clusters deposited as single-layer islands (isolated clusters, flat on the surface), whereas Pd_16_ through Pd_25_ showed signs that a second layer was beginning to appear to form (isolated clusters with some 3D structure).

The final state effects included the decrease of core-hole screening in small particles relative to bulk metals and the size-dependent charging energy of clusters on an insulating support. This charging energy would scale as *n*^−s^. The change in the scaling exponent S from 0.33 to 0.2 was expected to result from charge delocalization to the TiO_2_ surface in the final state, or from the deviation of the cluster shape from the spherical one. The binding energies fluctuated, and as shown below, these fluctuations were strongly correlated with reactivity. From the ISS and CO oxidation activity results, the onset of second-layer formation was correlated with increased activity for Pd_16_ and Pd_20_. On the other hand, although Pd_25_ continued the trend toward increasing second-layer filling, its reactivity was low. The BE for Pd_20_/TiO_2_ reached that for bulk Pd. ISS showed only the beginning of a second layer for the largest clusters, i.e. Pd cannot be considered to be in a bulk-like environment. Furthermore, the BE increased again substantially for Pd_25_. This pattern reflected the dependence of the initial- and final-state effects on the cluster size, and these effects incidentally cancelled each other for Pd_20_, resulting in a BE near the bulk limit. Final-state effects (charge localization and reduced screening compared with bulk Pd) tend to shift the BEs to higher energy. To cancel this shift, there must be an initial state that shifts to lower the BE, such as a net transfer of electron density from the TiO_2_ support to the Pd clusters. This electron transfer to the clusters also tends to have substantial effects on the ability of clusters to activate O_2_, which is presumably the limiting step in CO oxidation.

**Figure 5.**
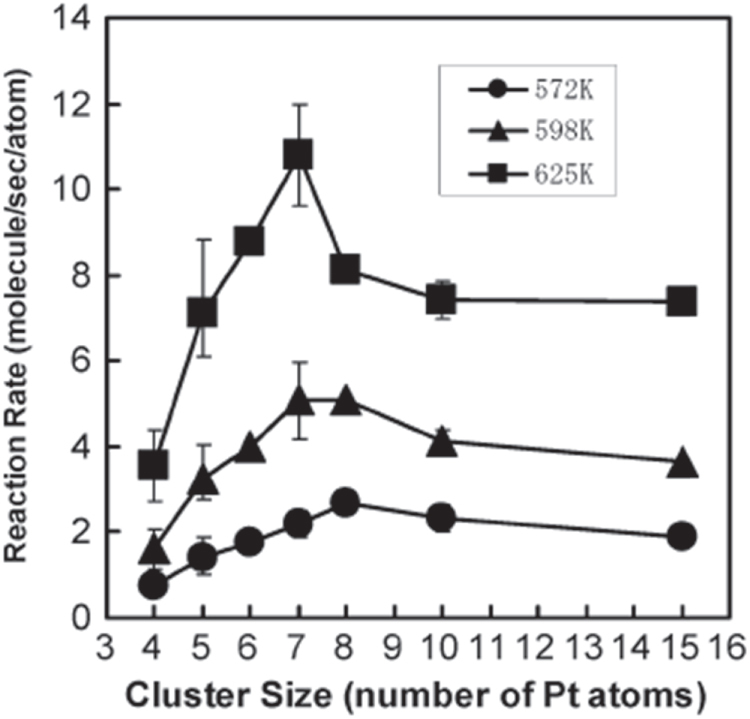
CO oxidation activity on Pt_*n*_/TiO_2_(110) (*n* = 4–8, 10, 15). (Reprinted with permission from [38], © The Royal Society of Chemistry 2011.)

**Figure 6.**
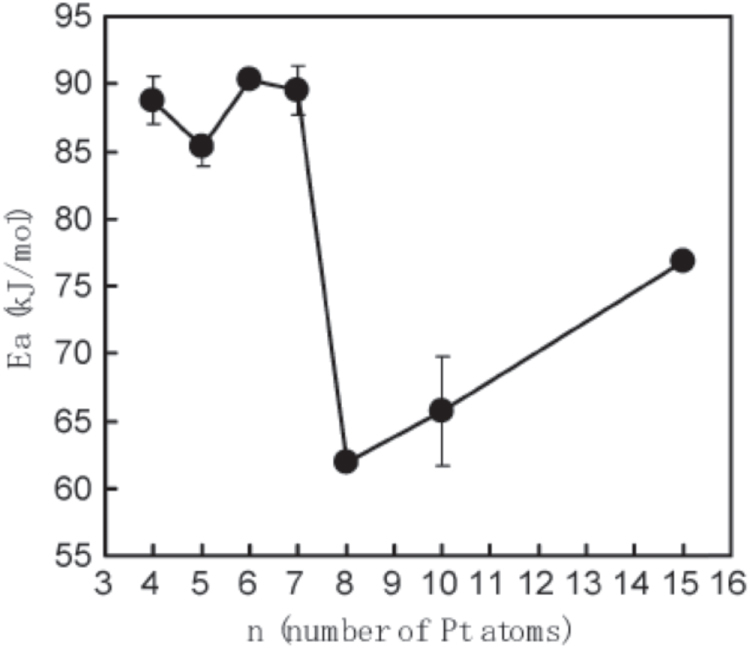
CO oxidation activation energy (*E*_a_) of Pt_*n*_ (*n* = 4–8, 10, 15)/TiO_2_(110) as a function of cluster size. (Reprinted with permission from [38], © The Royal Society of Chemistry 2011.)

We previously reported that the size dependence of Pt_*n*_ clusters supported on TiO_2_(110), as shown in figure 5 [38]. The catalytic oxidation of CO on this surface was investigated using a high-pressure reaction cell. The normalized production rates of CO_2_, relative to the number of Pt atoms in the sample, depended on the cluster size. The reactivity increased up to Pt_8_ at each temperature of 572 K and 598 K, and reached its maximum at 625 K when *n* = 7. The catalytic activity and activation energy shifted to a more active state at the same cluster size at which the planar-to-3D transition occurred, as shown in figure 6 [38]. A geometrical transition from a planar structure to a 3D structure was observed when the cluster size increased to 8 Pt atoms. The geometry of size-selected Pt clusters deposited on TiO_2_(110) was highly dependent on the number of atoms in the deposited cluster. The structural transition caused a shift to a more active state, presumably owing to Pt–Pt and Pt–surface interactions. The presence of a second layer of atoms in a Pt cluster on TiO_2_(110) may be associated with a significant decrease in the activation energy for CO oxidation. The second-layer Pt atoms were low-coordinated and interacted with the TiO_2_ surface indirectly.

**Figure 7.**
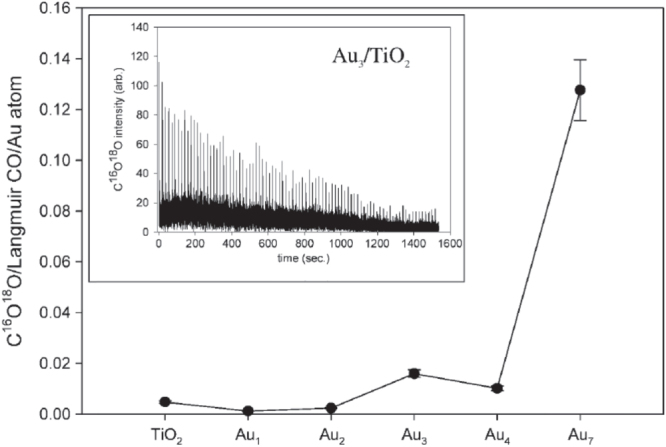
Size dependence of CO oxidation activity. Inset: effects of 100 CO pulses on Au_3_/TiO_2_. (Reprinted with permission from [39], © 2004 American Chemical Society.)

Anderson *et al* [39, 40] studied CO oxidation on size-selected clusters of Au_*n*_ (*n* = 1–7) supported on TiO_2_(110), as shown in figure 7. The activity at room temperature was studied using a pulse-dosing technique in which the catalyst is pre-dosed with O_2_ before exposure to short pulses of CO. Activity was correlated with the ability of the clusters to bind O_2_ and not with CO binding or cluster morphology.

It should be added that the influence of the surface oxygen vacancies on TiO_2_(110) were also investigated [41]. Pt_7_ clusters on a strongly reduced TiO_2_(110) surface were quenched in the CO oxidation.

CO oxidation activity has been organized by several key factors, including electronic states, electron transfer and cluster geometry resulting from cluster–support interactions. Size-dependent cluster geometry and corresponding catalytic activity was confirmed in the result of Pt_*n*_/TiO_2_(110) and Pd_*n*_/TiO_2_(110), but not in Au_*n*_/TiO_2_(110).

It also should be added that Lopez-Acevedo *et al* [42] have reported that electronic quantum size effects, particularly the magnitude of the so-called HOMO–LUMO (highest occupied molecular orbital and lowest unoccupied molecular orbital, respectively) energy gap, played a decisive role in binding oxygen to activate with low activation barriers from the result of ligand-protected gold clusters.

### 2.2. NO–CO reaction

The NO–CO reaction (2CO + 2NO → 2CO_2_ + N_2_) is one of the most important reactions for automotive exhaust purification, although only a few reports are available for this reaction.

**Figure 8.**
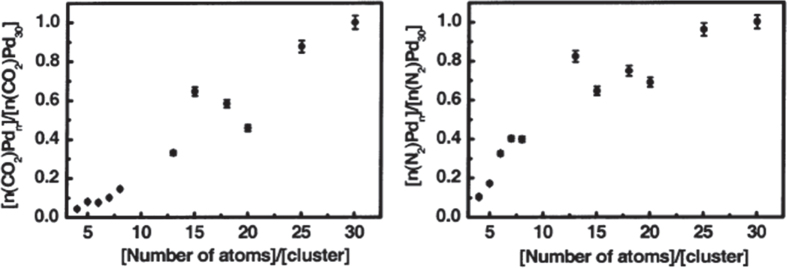
Reactivities expressed as the number of product molecules formed per cluster and normalized to the reactivity of Pd_30_/MgO film for ^13^CO_2_ and ^15^N_2_ as a function of cluster size. (Reprinted with permission from [43], © 2003 American Chemical Society.)

**Figure 9.**
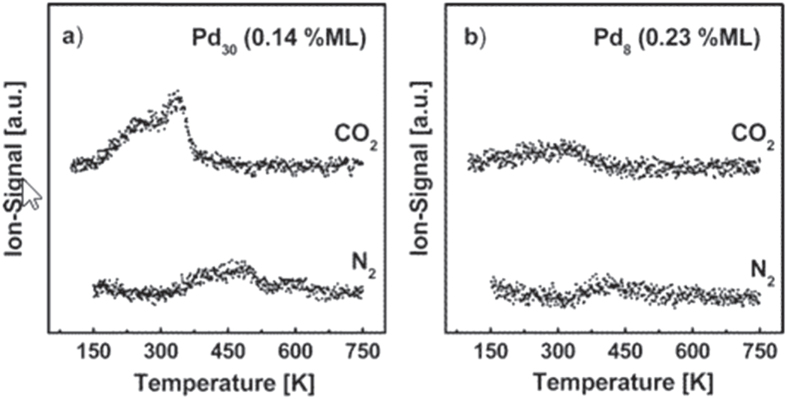
TPR results of an experiment in which Pd_30_ (a) and Pd_8_ (b) were first exposed to ^15^NO and subsequently annealed up to around 350 K. After the samples were cooled to 90 K, they were exposed to ^13^CO, and the resulting product molecules were monitored by TPR. (Reprinted with permission from [43], © 2003 American Chemical Society.)

Heiz *et al* [43] studied this reaction on size-selected Pd_*n*_ (*n* = 4, 5, 8, 15, 20, 30) clusters supported on MgO, as shown in figure 8. Clusters up to Pd_3_ were inert, while those up to Pd_19_ only showed reactivity at 300 K. Larger clusters were reactive at temperatures as low as 140 K. The high-temperature reaction mechanism involved the oxidation of CO by adsorbed oxygen atoms, whereas the low-temperature mechanism involved the direct reaction of CO with molecularly adsorbed NO. The efficiency of the reaction increased non-monotonically with cluster size, revealing a local maximum for Pd_15_, and a local minimum is observed for Pd_20_, as shown in figures 8 and 9 [43].

Further and detailed studies should be conducted regarding this reaction.

### 2.3. Acetylene cyclotrimerization

Acetylene cyclotrimerization to form an aromatic compound (benzene) is an important reaction for the chemical industry.

**Figure 10.**
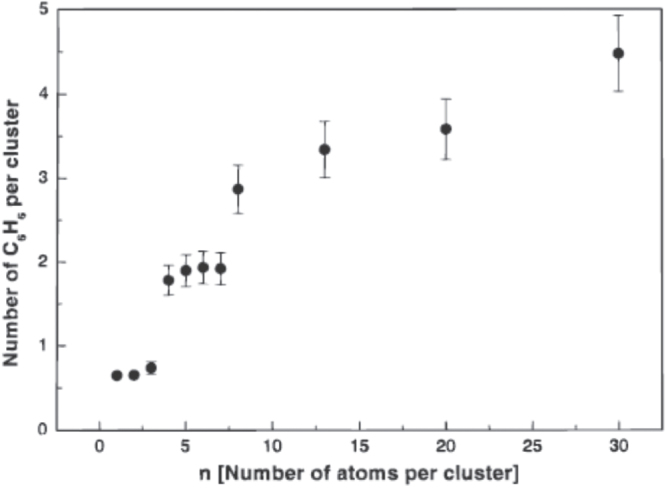
Total number of catalytically produced C_6_H_6_ molecules per cluster on Pd_*n*_/MgO(100) estimated by the integral of the TPR spectra and the number of deposited clusters. (Reprinted with permission from [44], © 2000 American Chemical Society.)

Heiz *et al* [44] studied the cyclotrimerization of acetylene on size-selected Pd_*n*_ clusters (*n* = 1–8, 13, 20, 30) supported on MgO(100) films, as shown in figure 10. Up to Pd_3_, benzene was exclusively produced at 300 K, whereas for Pd_7_, the formation of benzene began at about 430 K. It was suggested this additional product formation at 430 K for larger clusters (7 ≤ *n* ≤ 30) implied the existence of a critical ensemble of seven Pd atoms for the high-temperature reaction mechanism.

**Figure 11.**
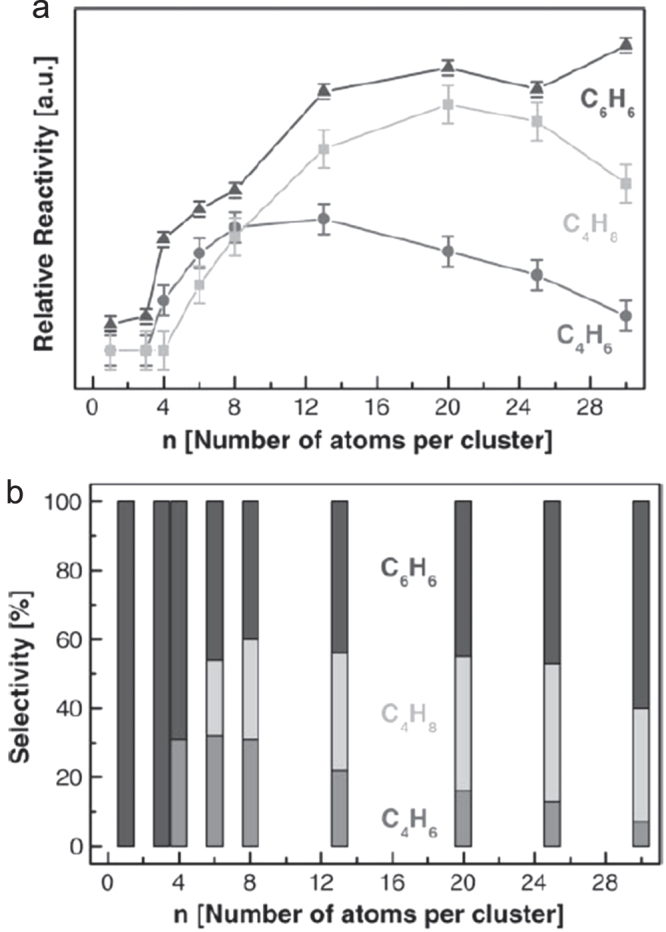
(a) Cluster-size-dependent reactivity expressed as the relative number per cluster of formed product molecules C_6_H_6_, C_4_H_8_, and C_4_H_6_. Maximal reactivity for the formation of C_4_H_6_ for cluster sizes around Pd_10_; for the formation of C_4_H_8_ for cluster sizes around Pd_20_, the formation of C_6_H_6_ increases with size. (b) Size-dependent selectivity in % for the polymerization of acetylene (see text). Pd_1–3_ show 100% selectivity for the cyclotrimerization, whereas the selectivity for the hydrogenation of the intermediate (C_4_H_4_) to C_4_H_6_ (30%) and C_4_H_8_ (≫40%) is maximal for Pd_6_ and Pd_20–25_, respectively. (Reprinted with permission from [45], © 2001 Academic Press.)

Acetylene polymerization was studied by TPR on well-defined model catalysts fabricated by soft landing of size-selected Pd_*n*_ (1 ≤ *n* ≤ 30) clusters on MgO(100) thin films. In a single-pass heating cycle experiment, C_6_H_6_, C_4_H_6_ and C_4_H_8_ were formed with product selectivities being dependent on the cluster size; Pd atoms selectively produced C_6_H_6_, while the highest selectivity for C_4_H_6_ and C_4_H_8_ was observed for Pd_6_ and Pd_20_, respectively, as shown in figure 11. These results provided an atom-by-atom observation of the selectivity of Pd_*n*_ model catalysts, which can be extended toward the cluster size on actual Pd catalysts, where a structure sensitivity for the cyclotrimerization was observed [45].

The size-dependent selectivity may be understood by the cluster size affect to steer the reaction either toward the cyclotrimerization (C_6_H_6_) or toward a direct hydrogen transfer (C_2_H_2_ to C_4_H_6_ and C_4_H_8_, via C_4_H_4_ intermediate). The key factors are the charge transfer related to the C–H bond activation and the pure geometry of Pd_*n*_ clusters. They implied it would be possible to tune atom by atom the activity and selectivity of actual catalysts.

### 2.4. Hydrazine decomposition

Hydrazine decomposition on metal surfaces is an important reaction for several industrial catalytic processes, including those requiring monopropellant thrusters and gas generators.

**Figure 12.**
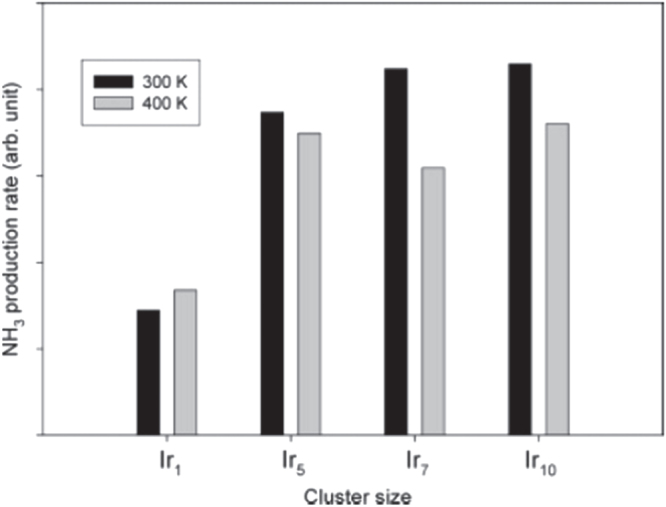
Relative NH_3_ production per deposited Ir atom as a function of cluster size and for 300 and 400 K surface temperatures, respectively. (Reprinted with permission from [46], © 2005 American Chemical Society.)

Anderson *et al* [46] studied hydrazine decomposition over a temperature range 0f 100–800 K for a series of model catalysts prepared by mass-selected Ir_*n*_^+^ deposition on Al_2_O_3_/NiAl(110). Temperature-programmed desorption (TPD) was used to study hydrazine desorption and decomposition on Al_2_O_3_/NiAl(110) and on the model catalyst prepared by deposition of Ir^+^ on Al_2_O_3_/NiAl(110) at a high density (5 × 10^14^ cm^−2^) as Ir_*n*_ cluster formation would be expected. The hydrazine decomposition activity of this model catalyst was found to be qualitatively similar to those observed on single-crystal Ir and polycrystalline Rh. A substantial decrease in the Ir XPS intensity suggested that considerable sintering occurred when the samples were heated to 800 K. In addition, a significant fraction of the hydrazine nitrogen was converted to an aluminium nitride (or mixed Al_*x*_O_*y*_N_*z*_) compound. Continuous flow experiments were used to probe relative reactivities at 300 and 400 K for samples prepared by depositing differently sized Ir_*n*_^+^ clusters. At 300 K, samples prepared with pre-formed Ir_*n*_^+^ (*n* = 5, 7, 10) are about twice that of samples prepared with Ir^+^ deposition. There was also a weaker trend toward higher activities with increasing cluster size, especially at 400 K, suggesting thermal modification of the samples, as shown in figure 12.

### 2.5. Cyclohexene aerobic oxidation

Partial oxidation reaction of hydrocarbons is an important reaction in the organic industry. For aerobic oxidation reaction of cyclohexane, both oxidation products, cyclohexanol and cyclohexanone, are the main industrial precursors of, respectively, *∊*-caprolactam and adipic acid, the building blocks of the nylon-6 and nylon-6,6 polymers.

**Figure 13.**
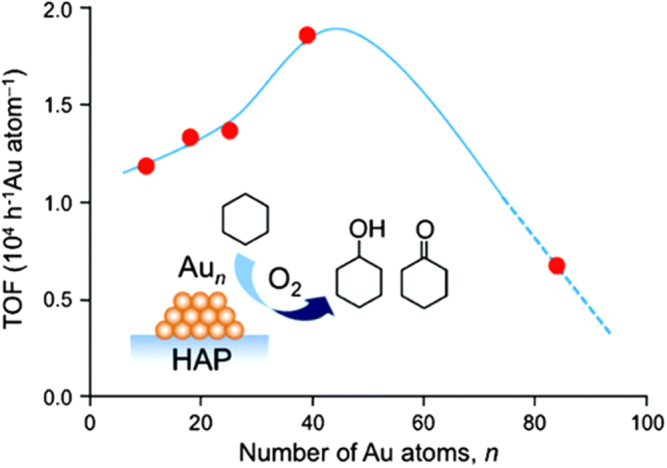
TOF values of Au_*n*_ clusters on hydroxyapatite (HAP) as a function of the cluster size *n*. The curve is a guide for the eye. (Reprinted with permission from [47], © 2010 American Chemical Society.)

Tsukuda *et al* [47] studied the catalytic aerobic oxidation of cyclohexene to cyclohexanol and cyclohexanone by Au_*n*_ (*n* = 10, 18, 25, 39) on hydroxyapatite (HAP). Cluster sizes were atomically controlled and found to influence the turnover frequency (TOF), which increased monotonically up to a value of 18 500 h^−1^ Au atom^*−*1^ at *n* = 39. However, the TOF subsequently decreased at higher *n* (up to ∼85), as shown in figure 13. The reactions were carried out under a 1 MPa O_2_ atmosphere at 423 K for 4 h. Selectivity to cyclohexanol and to cyclohexanone was about 50% and 50%, respectively. The author suggested that this finding could provide an important insight into size-specific catalysis by gold clusters (diameter <2 nm) and serve as a guide for the rational use of these catalysts.

### 2.6. Photocatalytic reaction

Heterogeneous photocatalysis which activates the chemical conversion using photonic energy has been studied in recent decades. The major applications of photocatalytic oxidation reaction are environmental pollution remediation, self-cleaning, and self-disinfecting. Numerous applications have been developed from utilizing photocatalytic reactions. The major applications of photocatalytic reductions are a conversion of water to hydrogen gas by photocatalytic water splitting and conversions of CO_2_ to formaldehyde (HCHO), formic acid (HCOOH), methyl alcohol (CH_3_OH), and methane (CH_4_).

**Figure 14.**
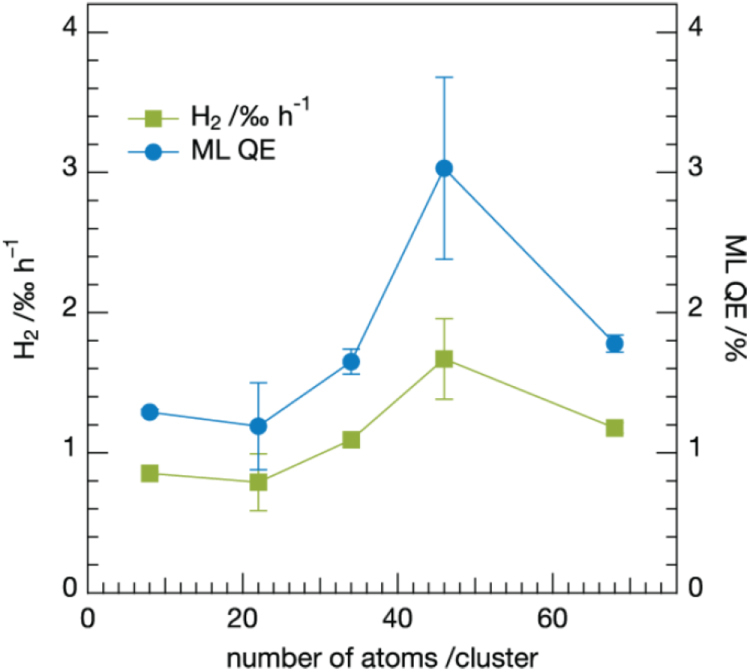
Photocatalytic activity of the CdS nanorods decorated with size-selected Pt clusters as a function of cluster size. The average H_2_ production rate clearly changes with the size of the clusters deposited. While a value below 1‰ h^−1^ is obtained for smaller clusters (Pt_8_ and Pt_22_), this value increases to over 1‰ h^−1^ for Pt_34_. After having reached a maximum H_2_ production rate for Pt_46_, the H_2_ production rate drops again for the larger clusters of Pt_68_. This trend is even more pronounced for the monolayer quantum efficiency (ML-QE) of the cluster–semiconductor catalysts, which takes into account that just the uppermost layer of the CdS nanorod film is covered with clusters. (Reprinted with permission from [48], © 2013 American Chemical Society.)

The effect of the Pt cluster size on photocatalytic water reduction on CdS nanorods was studied with maximum H_2_ production achieved with Pt_46_, as shown in figure 14 [48]. This effect was attributed to size-dependent electronic properties of the clusters; for example, the LUMO must be lower in energy than the lower edge of the conduction band of the semiconductor. They estimated the minimum amount of catalyst required to achieve maximum quantum efficiency for the reaction. It was also demonstrated that photocatalytic activities could be tuned through precise control of the cluster size of the catalyst [48]. Pt_46_ at different coverages and Pt_*n* ≥ 36_ (unselected and narrow distribution with *n* ≥ 36) clusters deposited on CdS nanorods showed significant H_2_ generation, while Pt_*n*≥36_ clusters on a blank substrate did not [48].

All samples containing both CdS nanorods and Pt clusters showed significant H_2_ generation, while Pt_*n*≥36_ clusters on a blank substrate did not. Figure 14 displays the H_2_ generation rate and monolayer quantum efficiency (ML-QE) of CdS nanorods covered with either Pt_*n*≥36_ or Pt_46_ clusters as a function of cluster coverage. The amount of generated H_2_ and ML-QE were low for coverages below 15 clusters/nanorod. Between 15 and 30 clusters/nanorod, both quantities sharply increased until a saturation point was reached. The catalytic activity of Pt_46_ was higher than the size-averaged one of the Pt_*n*≥36_ clusters, which demonstrated that differently sized clusters have different catalytic activities, and catalytic activity is determined by the precise number of atoms in the cluster.

**Figure 15.**
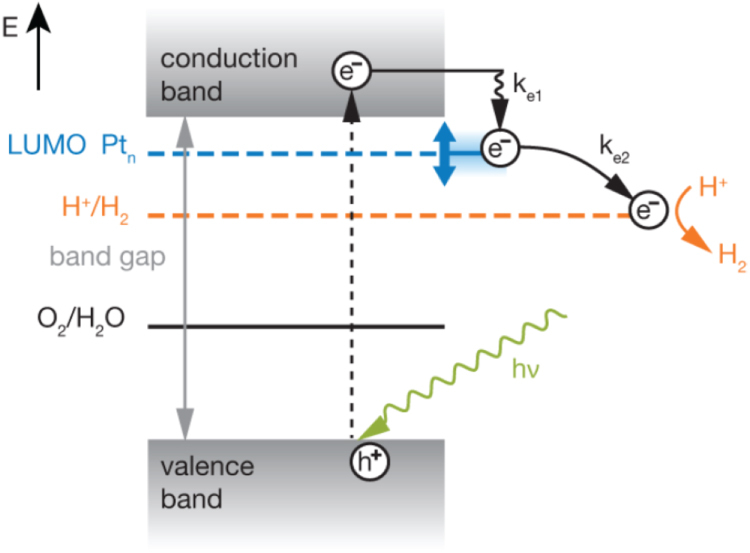
Reaction pathways for the photocatalytic H_2_ evolution. Under illumination with a photon with an energy larger than the band gap, an electron/hole pair is created in the semiconductor. In order to form H_2_, the electron has to be trapped efficiently at the cluster (*k*_*e1*_), and then transferred to the H^+^ atoms (k_e2_). For the formation of H_2_, two electrons have to be transferred to the protons. For an efficient trapping of the electron on the cluster, its LUMO has to be lower in energy than the lower edge of the conduction band of the semiconductor. Electron transfer from the cluster to H^+^ is energetically favored, if the cluster’s LUMO is higher in energy than the H^+^ reduction potential. Therefore, H_2_ evolution only takes place efficiently if the LUMO is positioned at an energy that is between the lower edge of the conduction band and the H^+^/H_2_ potential. An optimum position of the cluster’s LUMO is governed by these opposing effects. Tuning the LUMO by cluster size, a maximum in the photocatalytic activity is achieved with a certain number of atoms. (Reprinted with permission from [48], © 2013 American Chemical Society.)

Figure 15 displays the presumed reaction pathways for the photocatalytic H_2_ evolution [48]. This model suggests there exists an optimal LUMO energy level of clusters relative to the lower edge of the conduction band of the semiconductor and the chemical potential of the H^+^/H_2_ partial reaction by varying the cluster size. Furthermore, this can also be done for water oxidation, in which case, the occupied electronic states (HOMO) of the clusters should be between the upper edge of the valence band and the chemical potential of the O_2_/H_2_O partial reaction. Thus, the complete photocatalytic water splitting reaction may eventually become accessible through studies on the cluster size and coverage dependence of photocatalytic activities, intermediate and transition states of reactions, and charge carrier dynamics. The latter gives atomic-scale insight into these important reactions. This will be of crucial importance to the understanding and design of future photocatalytic nanosystems.

### 2.7. Electrochemical reaction

#### 2.7.1. Oxygen reduction reaction (ORR)

The oxygen reduction reaction (ORR: O_2_ + 4 H^+^ + 4e^−^ → 2H_2_O) is a key process in electrochemistry, especially in energy converting systems such as fuel cells. Recent progress in electrochemistry using size-selected clusters has been made by the several research groups [49–51].

Anderson *et al* [51] studied the ORR activity of Pt_n_/glassy carbon electrodes (Pt_*n*_/GCE) prepared by deposition of mass-selected Pt_*n*_^+^ (*n* ≤ 11) on GCE substrates. Electrocatalysis under appropriate reaction conditions was studied, for samples both in situ with no exposure to laboratory air and with air exposure prior to electrochemical measurements. Only a few cluster sizes showed the expected ORR activity, in which case, the activity/Pt atom was similar to that of a conventionally prepared electrode with Pt nanoparticles grown on the GCE under identical conditions. In the case of small clusters, the ORR signal was overwhelmed by large oxidative currents attributed to catalysis of carbon oxidation by water. Prior exposure of samples to air resulted in the disappearance of both ORR and carbon oxidation signals. Only small capacitive currents or currents attributed to the redox chemistry of adventitious organic adsorbates were observed, indicating that air exposure resulted in the passivation of the small Pt clusters.

**Figure 16.**
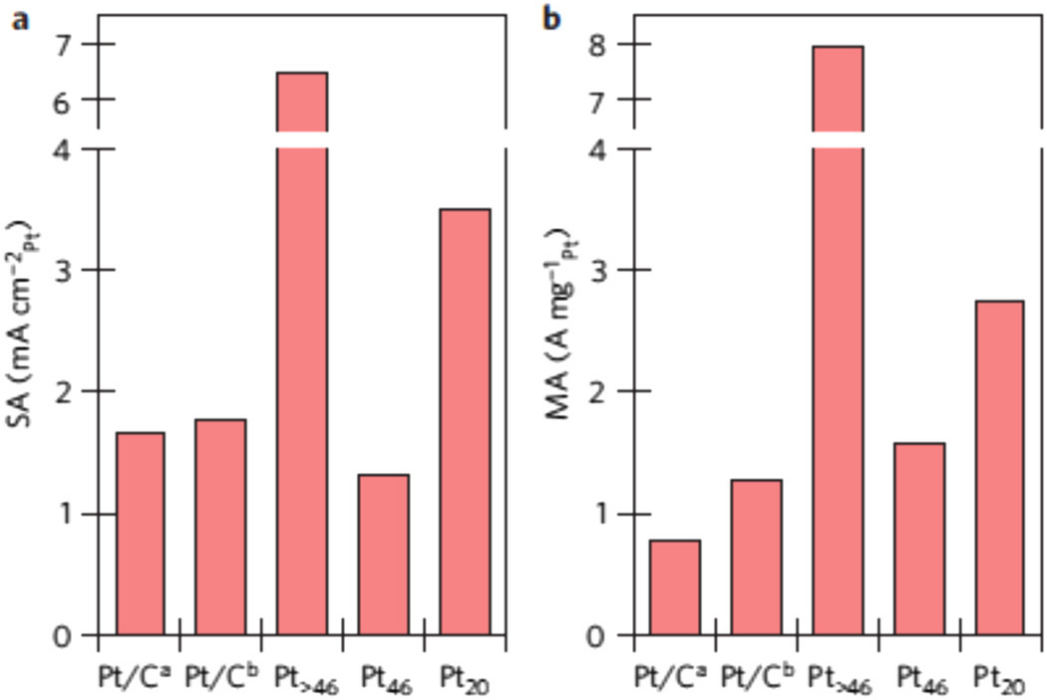
Maximum achieved ORR activities. SAs (a) and MAs (b) at 0.85 V_RHE_ for different Pt nanocluster samples. For comparison, the activities of two standard high-surface-area, carbon-supported Pt catalysts (TKK) are included as well. Both standard catalysts have a Pt loading of 50 wt.%; sample Pt/C^a^ has an average particle size of 5 nm, whereas the average particle size of sample Pt/C^b^ is 3 nm. The measurements were performed at room temperature in 0.1MHClO_4_ electrolyte. (Reprinted with permission from [53], © 2013 Macmillan Publishers Limited.)

Arenz *et al* [53] also studied the influence of cluster size and coverage on the ORR activity of Pt_*n*_/glassy carbon prepared by deposition of mass-selected Pt_*n*_^+^ (*n* = 20, 46, >46) on the planar substrate. In figures 16(a) and (b), the maximum achieved ORR surface-area-normalized specific activities (SAs) and mass specific activities (MAs), respectively, of different well-defined, size-selected Pt cluster samples (at 0.85 V_RHE_) are summarized. Note that the last Pt_>46_ (*d* = 2.3 nm) nanoclusters were deposited by operating the quadrupole mass spectrometer in the rf-only mode with a selected mass of Pt_46_ as a high-pass filter. The SA of Pt_20_ and Pt_>46_, was significantly enhanced by factors of 2 and 3.5, respectively, relative to that of standard catalysts. The MA of Pt_20_ and Pt_>46_, which is related to the expected power output per amount of Pt, also increased by factors of more than 2 and 6, respectively. No apparent correlation between the SA and the cluster size was observed, and Pt_>46_ showed the highest mass activities. The measured ORR SA of the Pt nanocluster samples instead depended on the edge-to-edge distance: this value increased significantly for various samples when the average distance became shorter than 1 nm, independent of the nanocluster size. These results implied that the catalytic activity depends not only on the cluster size, but also on the local configurations.

#### 2.7.2. Oxygen evolution reaction (OER)

**Figure 17.**
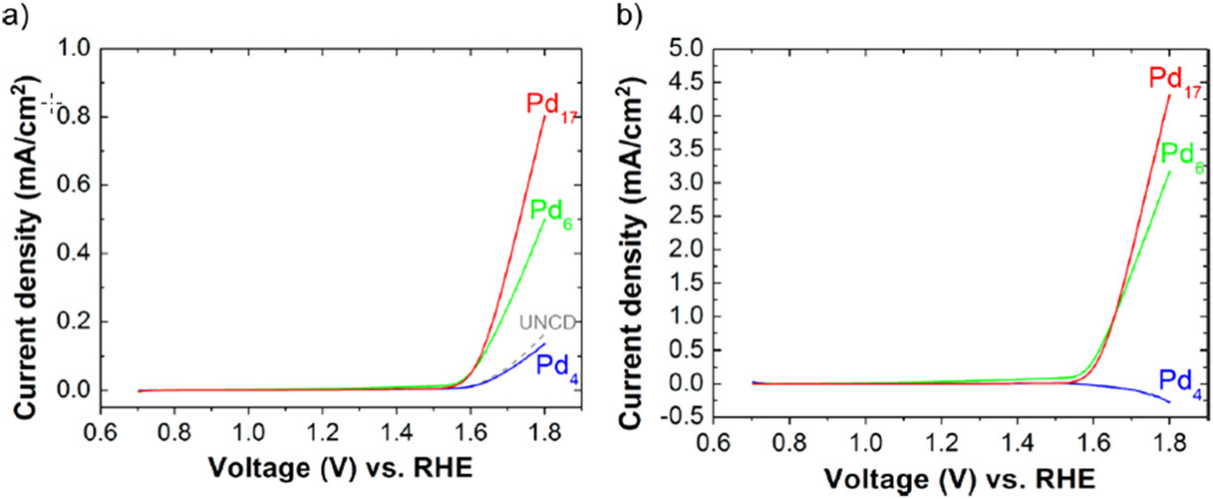
(a) Performance of size-selected palladium cluster on UNCD under pH 13.6 (1 M NaOH) with current densities normalized to the dipped chip area, *S*_chip_, for the blank UNCD support (gray, dashed line), Pd_4_ (blue), Pd_6_ (green), and Pd_17_ (red) clusters. The fraction of surface area, *S*_cluster_, covered by clusters was 9.4%, 7.9%, and 11.0% for the Pd_4_, Pd_6_, and Pd_17_ clusters, respectively, calculated as *S*_cluster_/*S*_chip x_100. (b) Background corrected (i.e., after subtraction of the current of the blank UNCD support) currents normalized for the cluster-covered area. (Reprinted with permission from [52], © 2013 American Chemical Society.)

Vajda *et al* [52] studied water oxidation in alkaline conditions using Pd clusters to probe the relationship between cluster size and the reaction, as shown in figure 17. There were no reactions with Pd_4_; however, the deposited Pd_6_ and Pd_17_ clusters were the most active in terms of turnover rate per Pd atom. Theoretical calculations suggested that this striking difference may indicate that bridging Pd–Pd sites (which are only present in 3D clusters) are active in the OER in Pd_6_O_6_. The ability to experimentally synthesize size-specific clusters allowed direct comparison to theoretical data. An ultrananocrystalline diamond (UNCD), which is sufficiently thin for electrical conduction and chemically/electrochemically very stable, was used as the support electrode. UNCD had a very wide working electrochemical potential window and showed only minor evidence of reaction. They remarked that the system (soft-landed Pd_4_, Pd_6_, or Pd_17_ clusters on a UNCD Si-coated electrode) had stable electrochemical potentials over several cycles, and synchrotron studies of the electrodes showed no evidence of evolution or dissolution of either the electrode material or the clusters, as shown in figure 17.

### 2.8. Computational chemical approach

Substantial computational studies have demonstrated the influence of the support material of clusters on heterogeneous catalysis [54–58]. However, such studies on size-specific catalytic reaction on size-selected clusters are scarce. Landman *et al* [32] reported a theoretical study on tuning the catalytic activity of Au nanoclusters via support design. The current state of understanding of various factors controlling the reactivity and catalytic activity of nanostructures was discussed using CO oxidation by Au nanoclusters adsorbed on MgO as an example. The role of the metal-oxide support and its defects, charge state, and structural fluxionality of the clusters, electronic size effects, effect of the underlying metal support on dimensionality, charging and chemical reactivity of Au clusters adsorbed on the metal-supported metal-oxide, and promotional effect of water were examined. Finally, a detailed picture of the reaction mechanism obtained through combined experimental and first-principles quantum mechanical calculations and simulations was proposed [32]. Activity was shown to originate from the dimensionality crossover of Au clusters: the 3D optimally structured Au clusters on thick MgO films were inactive, while 2D Au clusters on thin MgO films/Mo(100) were active. The underlying metal formed an electrostatic interaction with metal-induced excess electronic charge accumulated at the cluster interface with the metal-oxide film. This excess charge was predicted to activate O_2_ molecules adsorbed at the interfacial periphery of the 2D Au island with the MgO/Mo(100) surface by significantly weakening the O–O bond. This resulted in a remarkably lower barrier for the reaction with CO and subsequently weakened rather remarkably the barrier for reaction of the activated molecule with CO and the subsequent emission of CO_2_. The planar isomer was more stable than the 3D one by 3.3 eV owing to the penetration of metal states through the thin MgO film and charge accumulation at the cluster/MgO interface. Electronic charge transfer to the antibonding 2*π*∗ orbital of the O_2_ strongly adsorbed on the periphery of a planar Au_20_ cluster activated the O–O bond to the peroxo state with no spin polarization. From this study of reaction mechanisms, it was proposed that the reactivity could be controlled through manipulations of the supporting substrate. On the other hand, the interfacial charge can be controlled, for example, through the use of applied fields.

It was also noted that small clusters have structural isomers of comparable energies that can interconvert at finite temperature through (1) the formation of an equilibrium ensemble of coexisting structural isomers of the model catalyst, each exhibiting different chemical reactivities and (2) structural fluxionality, which is essential for the reaction to proceed as the cluster will no longer be constrained to the original geometry, thus preventing the adsorption and activation of O_2_ [32].

## 3. Conclusions and perspectives

In this review, we gave an overview of catalytic activity of metal clusters deposited on a support in light of atomically controlled sizes in relation to the type of reactions. A number of excellent studies was driven forward by several research groups. The results obtained so far are controversial. A few pieces of the huge puzzle are fitted.

We have reviewed a number of studies in this area. Not much is available on atomically controlled cluster catalysis with the exception of the CO oxidation reaction. Even CO oxidation reaction is still incompletely understood. Several lines of evidence indicated that the electronic structure of clusters and the geometry of clusters on a support, including the accompanying cluster–support interaction, are strongly correlated with catalytic activity. The electronic states of small clusters may easily be affected by cluster–support interactions. The catalytic activity can be enhanced by controlling not only the cluster size, but also the interactions between the clusters and the support material. This is an important practical advantage of atomically precise cluster catalysts to drastically reduce the utilization of precious or rare metals.

There are two main motivating forces in the field of atomically controlled cluster catalysis. One is to study the mechanism of catalysis and the other is to explore size-specific catalytic activity. Studies employing atomically precise clusters as well-defined model catalysts are continuously conducted with the goal of understanding the true nature of catalysis. With an atomically precise, controlled catalyst, it is reasonable to use these as model catalysts. Because we still have only patchy information, and many difficulties remain, it is necessary to continue making persistent efforts.

A few studies have focused on quantum effects on the catalytic properties of size-controlled clusters. Although several mechanisms have been suggested, these effects remain poorly understood. It has been proposed that in the studies reviewed in this paper that it is possible to tune the electronic structure through atomic control of the cluster size. In addition to so-called HOMO–LUMO energy gap, the hybridization between the electronic states of the adsorbed reactant molecules and cluster at *E*_F_ can be tuned to realize a quantum-controlled catalyst.

The preconceived notion that small clusters have a lower melting point has been influential. However, atomically precise clusters are easily and strongly affected by cluster–surface interactions. These interactions and monodispersity can lead to promising effects that will hinder the aggregation of such clusters on the surface. This is an important practical advantage to drastically reduce the utilization of precious or rare metals.

It is important to the understanding of these reactions to conduct experiments under repeated cycles such as a catalytic cycle and not under the TPR method, which is an irreversible process. This is because catalytic reactions should be considered under a catalytic cycle. Furthermore, the temperature dependence of reaction rates should be studied to estimate the activation energy, which is one of the most important factors for comparing the performance of catalysts.

There is one last point that is eagerly anticipated. Computational chemistry will move into the frontline and lead the way to the growth and development of atomically precise cluster catalysis towards quantum-controlled catalysts. Sophisticated software and hardware for computational chemistry and an accessible database specifically developed for the cluster research will be useful. Exchange of useful knowledge among experimental and computational scientists, particularly those working on cluster and surface chemistry, is also important. Finally, this field of research should be supported industry and governments across borders. In closing, it would be great help if the killer application for atomically precise cluster catalysis will be developed soon.
